# Targeting NOX2 and glycolytic metabolism as a therapeutic strategy in acute myeloid leukaemia

**DOI:** 10.1186/s40364-024-00674-x

**Published:** 2024-10-29

**Authors:** Carla Ijurko, Marta Romo-González, Rodrigo Prieto-Bermejo, María Díez-Campelo, María-Belén Vidriales, Sandra Muntión, Fermín Sánchez-Guijo, Jesús Sánchez-Yagüe, Ángel Hernández-Hernández

**Affiliations:** 1 Departamento de Bioquímica y Biología Molecular, Universidad de Salamanca, Plaza Doctores de la Reina, s/n, Salamanca, 37007 Spain; 2 IBSAL (Instituto de Investigación Biomédica de Salamanca), Salamanca, 37007 Spain; 3 Servicio de Hematología, Hospital Universitario de Salamanca, Salamanca, Spain; 4 Departamento de Medicina, Universidad de Salamanca, Salamanca, 37007 Spain; 5 Present Address: Present Address: Susan Lehman Cullman Laboratory for Cancer Research, Rutgers University, Piscataway, NJ 08854 USA; 6 Present Address: Present Address: Rutgers Cancer Institute, Rutgers University, New Brunswick, NJ 08901 USA

**Keywords:** Acute myeloid leukaemia, NADPH oxidase, NOX2, *CYBB*, Glycolysis, Hexokinase, Lactate dehydrogenase (LDH)

## Abstract

Acute myeloid leukaemia (AML) is a highly heterogeneous malignancy, with a poor 5-year overall survival rate of approximately 30%. Consequently, the search for novel therapeutic strategies is ongoing, and the identification of new vulnerabilities could accelerate progress. Oxidative stress and metabolic rewiring are established hallmarks of cancer, and recent evidence suggests that NADPH oxidases may regulate metabolism, potentially linking these two processes. Increasing research highlights the importance of NOX2 in AML, particularly its role in metabolic regulation. In this study, we investigated the effects of simultaneously inhibiting NOX2 and glycolysis in AML cells. Dual inhibition of NOX2 and glycolysis—by targeting hexokinase or lactate dehydrogenase (LDH)—significantly reduced cell proliferation, markedly impaired clonogenic potential, and induced extensive cell death in a broad panel of AML cell lines. Importantly, these findings were further validated in primary bone marrow samples derived from AML patients, where combined inhibition triggered similar potent anti-leukemic effects. Furthermore, the combined inhibition of NOX2 and LDH enhanced the efficacy of cytarabine (AraC), suggesting this approach could boost the effectiveness of conventional therapies. In an in vivo AML model, targeting NOX2 and LDH in myeloid progenitor cells delayed the onset of leukaemia and extended survival. In conclusion, our findings propose a novel therapeutic strategy for AML through the dual targeting of NOX2 and glycolysis.

**Supplementary Information:**

The online version contains supplementary material available at 10.1186/s40364-024-00674-x.

To the editor,

Acute myeloid leukaemia (AML) is the most common form of acute leukaemia in adults, with a poor prognosis and a 5-year overall survival rate of about 30% [1]. The “3 + 7” regimen of anthracycline and cytarabine (AraC) has been the standard since the 1970s [2]. Despite the introduction of new compounds into clinical practice, improvements remain insufficient [3].

NADPH oxidases, a family of enzymes that produce reactive oxygen species (ROS), control metabolism and may link oxidative stress and metabolic rewiring, both key features of cancer exploitable for therapy [4]. Previous evidence underscores the importance of NOX2 in AML. NOX2 activity has been linked to glycolysis activation in AML [5, 6] and to mitochondria transfer from stromal to leukemic blasts [7]. Additionally, NOX2 serves as a prognostic marker in AML [8, 9] and may also be a viable therapeutic target [10, 11].

We hypothesised that targeting NOX2 and metabolism could uncover new therapeutic strategies for AML. In this study, we analysed the effect of simultaneously inhibiting NOX2 and glycolysis on cell lines and primary AML patient cells, investigated the impact on leukaemia onset in vivo, and evaluated the potential to enhance chemotherapy effects.

To target glycolysis, we used 2-Deoxy-D-Glucose (2-DG), a glucose analogue inhibiting hexokinase, and oxamate, an inhibitor of lactate dehydrogenase (LDH) (Fig. S1). Combined with diphenyleneiodonium (DPI), a common NADPH oxidase inhibitor, these agents significantly reduced cell proliferation in a synergistic manner (Fig. 1A-B). Notably, the combination of DPI and oxamate demonstrated a profound inhibitory effect, resulting in almost complete inhibition of cell growth (Fig. 1A-B) and clonogenic potential (Fig. 1C-D), alongside a massive induction of cell death (Fig. 1E-F). This synergistic effect was further validated using alternative inhibitors for NADPH oxidases (perphenazine and apocynin) and LDH (FX-11) (Fig. S2).

**Fig. 1:**
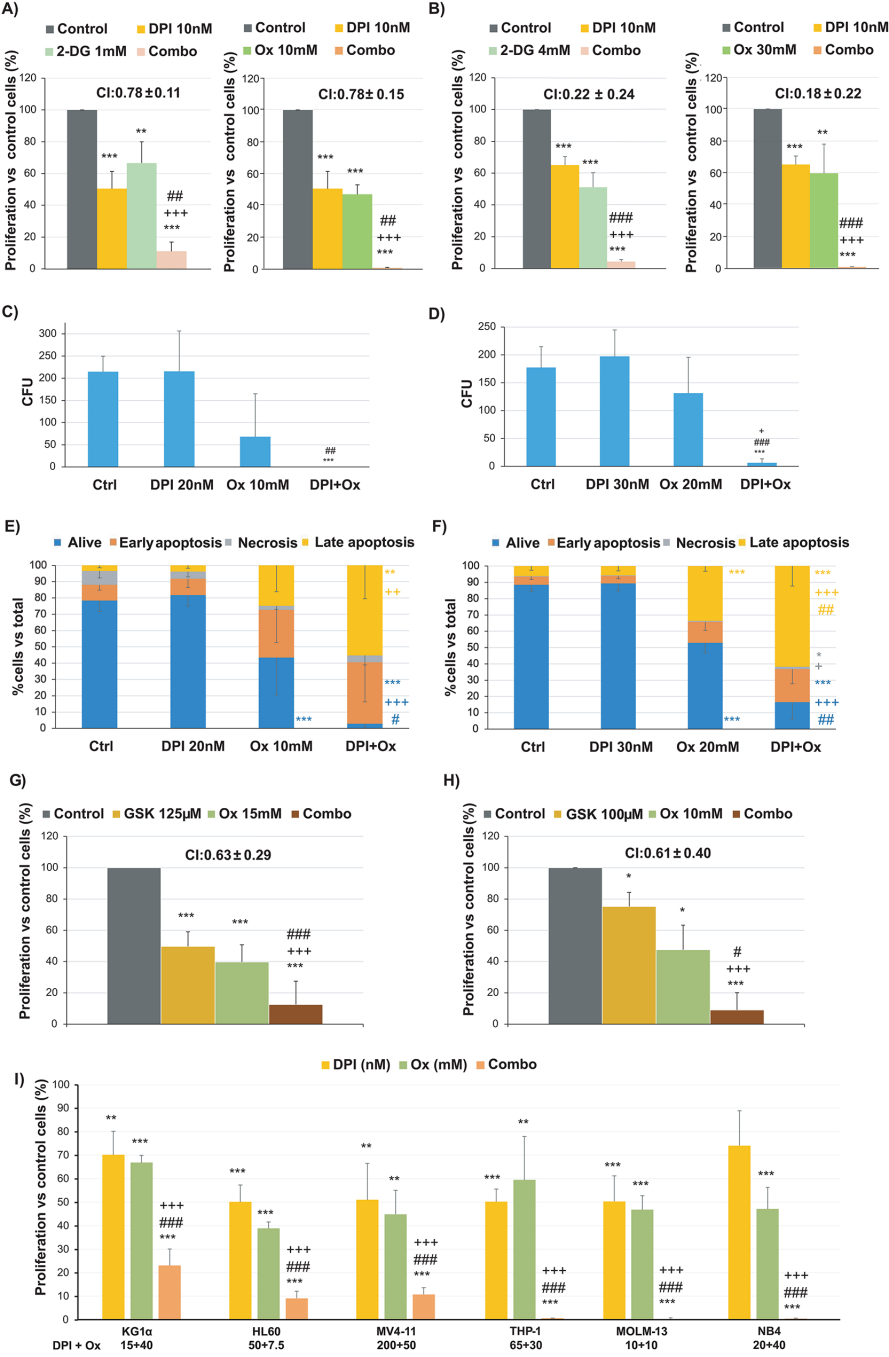
Treatment with inhibitors of NADPH oxidases and glycolysis synergistically inhibits proliferation, viability, and colony-forming ability in AML cell lines. AML cell lines were cultured for 48 h in the presence or absence of varying concentrations of 2-DG (hexokinase inhibitor), Ox (LDH inhibitor), DPI (pan-NOX inhibitor), GSK2795039 (NOX2-specific inhibitor), or their combinations. **A**-**B**) Cell proliferation rates relative to untreated control cells were evaluated in MOLM-13 (**A**) and THP-1 (**B**) cell lines (*n* = 6). The combination index (CI) for each inhibitor combination is shown on the graph. **C**-**D**) Number of colony-forming units (CFU) counted after 7 days of culturing 500 MOLM-13 cells (**C**) or THP-1 cells (**D**) in 500 µl of semisolid methylcellulose medium (*n* = 5). **E**-**F**) Percentages of live cells (Annexin-/7AAD-), cells in early apoptosis (Annexin +/7AAD-), late apoptosis (Annexin +/7AAD+), and necrosis (Annexin-/7AAD+) in MOLM-13 cells (**E**) and THP-1 cells (**F**) were determined by Annexin/7AAD staining (*n* = 6). **G**-**H**) Cell proliferation relative to untreated control cells was evaluated for Ox, GSK2795039, or their combination in MOLM-13 (**G**) and THP-1 (**H**) cell lines. Each combination was assessed in at least 3 experiments. The combination index (CI) is indicated for each inhibitor combination. **I**) The cell proliferation rate compared to untreated control cells of Ox + DPI combination was assessed in KG1α (derived from undifferentiated AML, M0, characterised by chemotherapy resistance); HL60 (M2); MV4-11 (M5, positive for *FLT3-ITD* mutation); NB4 (M3 positive for *PML*-*RAR* translocation); MOLM-13 (M5a, positive for *FLT3-ITD* mutation) and THP-1 (M5b positive for *MLL-AF9* translocation) cell lines. The numbers below each cell line indicate the concentration of DPI and Ox, respectively, used for each cell line. Proliferation experiments were performed at least 4 times. ***p < 0.001, **p < 0.01 and *p < 0.05 reflect significant differences compared to untreated control cells. +++*p* < 0.001, ++*p* < 0.01 and + *p* < 0.05 reflect significant differences compared to cells treated with NOX inhibitor, DPI or GSK2795039, alone. ###*p* < 0.001, ##*p* < 0.01 and #*p* < 0.05 reflect significant differences compared to cells treated with metabolism inhibitor (Met Inh.), 2-DG or Ox. 2-DG, 2-Deoxy-D-Glucose; Ox, oxamate

Given that NOX2 is the main NADPH oxidase isoform in AML cells [12], the observed effects can be attributed to NOX2 inhibition. To test this hypothesis, we used GSK2795039, a NOX2-specific inhibitor. When combined with oxamate, this targeted AML cells in a synergistic manner (Fig. 1G-H). In contrast, no synergy was observed when oxamate was combined with GKT137831, which inhibits NOX1 and NOX4 (Fig. S3). Additionally, we tested the effect of DPI, oxamate and their combination in a panel of AML cell lines with varying levels of NOX2 expression [6]. Notably, as NOX2 expression increases (from left to right) [6], the inhibitory effect of the combination becomes more pronounced (Fig. 1I).

We also evaluated the impact on primary bone marrow cells obtained from AML patients. Treatment with DPI, 2-DG or oxamate, or their combination reduced cell proliferation (Fig. 2A) and colony-forming unit (CFU) activity (Fig. 2B), while increasing cell death (Fig. 2C). The combined treatment exhibited a more pronounced effect, particularly in reducing cell proliferation (DPI + 2-DG) and inducing cell death (DPI + oxamate), which was statistically stronger than the effect observed with single treatments. Comparable results were obtained with GSK2795039 combined with 2-DG (Fig. S4A-B). Applying the same approach to different cell populations from healthy donors (Fig. S4D), we found that the dual treatments did not elicit a meaningful increase in cell death relative to the single treatments, except for the DPI and oxamate combination in monocytes (Fig. S4E-H). This finding strengthens the translational potential of our results.

**Fig. 2:**
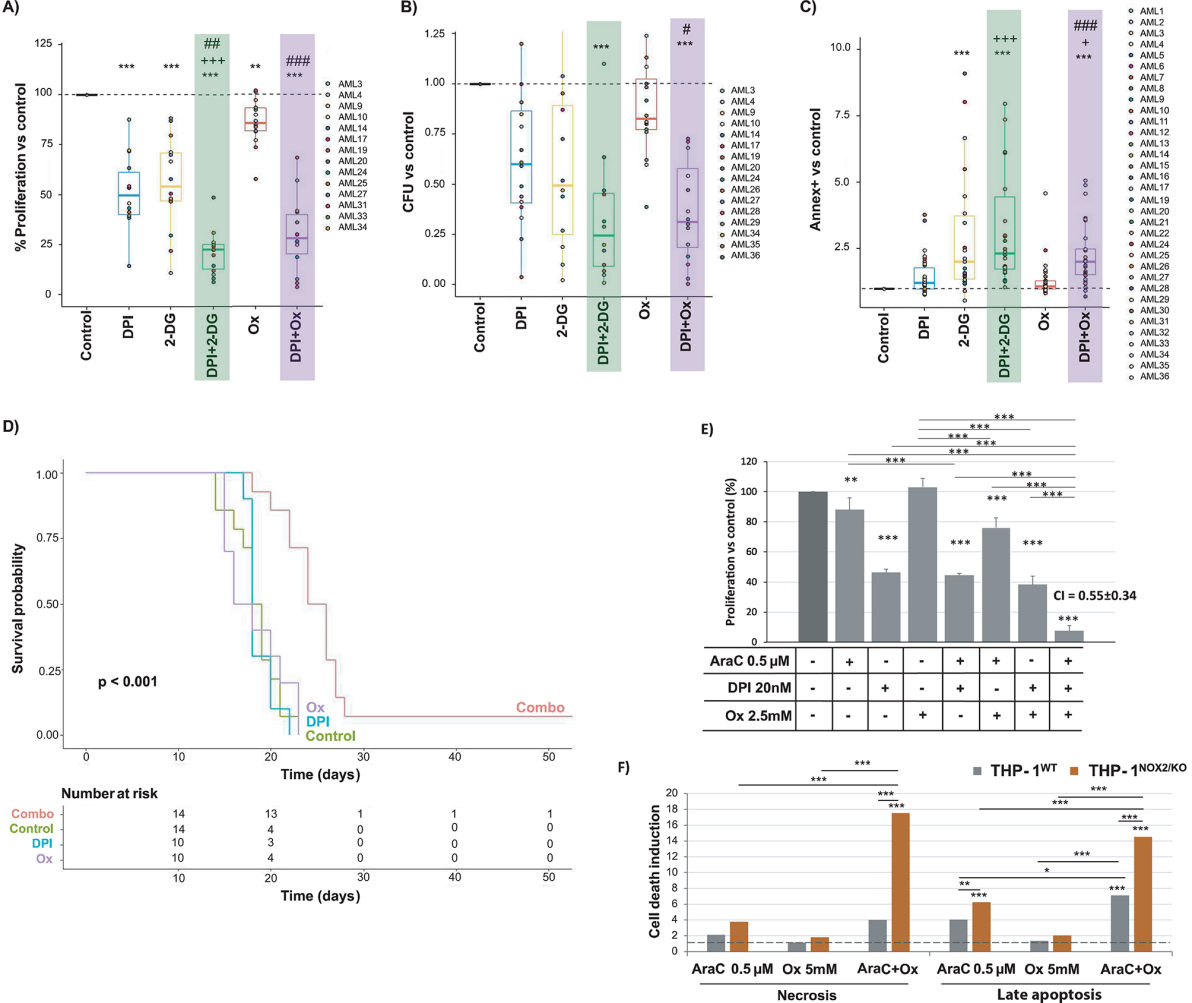
The combination of glycolysis inhibitors with NOX inhibitors demonstrates translational potential by effectively treating AML patient cells in vitro, prolonging survival in an in vivo leukaemia model, and enhancing cytarabine cytotoxicity. **A**-**C**) Mononuclear cells extracted from the bone marrow of various AML patients using a Ficoll gradient were cultured at a density of 5 × 10^5^ cells/ml for proliferation assays and 1 × 10^6^ cells/ml for clonogenicity and cell viability assays. The cells were cultured for 48 h in the presence of 2 mM 2-DG, 10 mM Ox, 100 nM DPI, or their combination. Cell proliferation was assessed by MTT assay when 2-DG or Ox was combined with DPI (**A**). Normalization of the number of colony forming units (CFU) relative to control counted after culturing 10^4^ mononuclear cells from different AML patients previously treated with 2-DG or Ox combined with DPI in 500 µl of semi-solid methylcellulose medium for two weeks was assessed (**B**). Cell death induction of Annexin + cells in the SSC^low^ CD45^low^ population was quantified by combining 2-DG or Ox with DPI (**C**). All graphs display data normalised relative to the control. **p < 0.001, **p < 0.01 and *p < 0.05 reflect significant differences compared to untreated control cells. +++*p* < 0.001, ++*p* < 0.01 and + *p* < 0.05 reflect significant differences compared to cells treated with DPI alone. ###*p* < 0.001, ##*p* < 0.01 and #*p* < 0.05 reflect significant differences compared to cells treated with metabolism inhibitor, 2-DG or Ox. **D**) Murine granulocyte-monocyte progenitors transformed with the *MLL-AF9* translocation were treated with 200nM DPI, 20mM Ox, or their combination in vitro for 24 h. The figure shows the overall survival of mice transplanted with either pre-treated or control granulocyte-monocyte progenitor cells. Day 0 represents the day of transplantation. **E**) THP-1 cells were cultured to assess cell proliferation by MTT assay after 48 h in the presence of cytarabine (AraC), Ox and DPI or their combination. The combination index (CI) is indicated for each inhibitor combination. **F**) Wild type and NOX2-deleted THP-1 cells and were cultured for 48 h in the presence or absence of 0.5µM AraC, 5mM Ox or AraC + Ox. Cell viability was analysed by annexin/7AAD staining, and the percentages of cells in necrosis (Annexin-7AAD+) and late apoptosis (Annexin + 7AAD+) are depicted in the figure (*n* = 5). ***p < 0.001, **p < 0.01 and *p < 0.05 reflect significant differences in E-F. 2-DG, 2-Deoxy-D-Glucose; Ox, oxamate

Next, we analysed the effect on the onset of AML in vivo using a murine model. Treatment of myeloid progenitor cells with the combination of DPI and oxamate prolonged animal survival (Fig. 2D), suggesting that the combined treatment is particularly effective against leukaemia-initiating cells.

Finally, we analysed the interaction with chemotherapy. No interaction was observed with daunorubicin (DNR) (Fig. S5A-B), but AraC exhibited positive interactions with DPI, GSK2795039, and oxamate (Fig. S5C-D). Furthermore, the triple combinations effectively inhibited cell proliferation more than the individual and double combinations (Fig. 2E). Additionally, genetic deletion of NOX2 significantly enhanced cell death induction by AraC and AraC + oxamate compared to wild-type cells (Fig. 2F), validating the synergistic effects observed throughout this study and highlighting NOX2 as a promising therapeutic target for AML.

In summary, our results offer an alternative therapeutic option for AML patients by targeting NOX2 and glycolytic metabolism, which also enhances chemotherapy efficacy.

## Electronic supplementary material

Below is the link to the electronic supplementary material.

Supplementary Material 1

## Data Availability

No datasets were generated or analysed during the current study.

## Abbreviations

AML: Acute myeloid leukaemia
AraC: Cytarabine
2-DG: 2-Deoxy-D-Glucose
DNR: Daunorubicin
DPI: Diphenyleneiodonium
LDH: Lactate dehydrogenase
NOX: NADPH oxidase
ROS: Reactive oxygen species
