# Supplementary material for: Targeting NOX2 and glycolytic metabolism as a therapeutic strategy in acute myeloid leukaemia
Source: Biomark Res. 2024 Oct 29;12:128. doi: 10.1186/s40364-024-00674-x (PMC11520369; doi:10.1186/s40364-024-00674-x)
Supplement: Supplementary file 1 — Supplementary Material 1 [file 40364_2024_674_MOESM1_ESM.pdf]

SUPPLEMENTARY MATERIAL AND METHODS

METHODS

Cell culture

All cell lines were cultured at 37ºC and 5% CO_2. Periodic testing for mycoplasma contamination was conducted using the PlasmoTest™ test (Invivo-Gen, France, cat #rep-pt1), following the manufacturer's instructions. Bone marrow mononuclear cells (BM-MNC) were obtained from AML patients at diagnosis at the University Hospital of Salamanca. In all cases, informed consent was obtained from each patient in accordance with the protocols approved by the local Ethics Committee.

Cell proliferation and drugs interaction analyses

Cell proliferation was assessed using the MTT assay (3-(4,5-dimethylthiazol-2-yl)-2,5-diphenyltetrazolium bromide), as we have done previously [1]. Drug interaction analysis was performed using the median-effect method, as outlined by Chou-Talalay [2]. The combination index (CI), computed using CalcuSyn software (Biosoft, Cambridge, UK), was utilized to determine the interaction between drugs, categorizing it as synergy (CI < 1), additivity (CI = 1), or antagonism (CI > 1).

Cell viability analyses

Cell viability was assessed using flow cytometry following staining with an Annexin V-PE/7-aminoactinomycin (7-AAD) detection kit (Immunostep, Salamanca, Spain),

according to the manufacturer's instructions. AML blasts were identified by staining with CD45 as the $SSC^{low}/CD45^{dim}$ region as reported [3].

Colony forming unit assays

Cell clonogenic capacity was assessed using colony-forming unit (CFU) assays in semisolid methylcellulose medium. AML cell lines or primary bone marrow mononuclear cells (BM-MNC) from AML patients were treated with the indicated drugs for 48 hours. After treatment, cells were washed with PBS and either 500 cells from AML cell lines or 10,000 BM-MNC cells were suspended in 500 µl of "HSC-CFU-basic" or "HSC-CFU-complete w/o Epo" medium, respectively (Miltenyi Biotec; Madrid, Spain), and seeded onto a culture plate. Cells were then incubated at 37ºC and 5% CO_2, and colonies were counted by blinded scoring at day 7 for AML cell lines, and at day 14 for primary samples.

Analyses of *in vivo* leukemic potential

A murine AML model driven by the *MLL-AF9* translocation [4, 5] was employed to assess the effects of the drugs on leukemic potential in vivo. Murine myeloid progenitors transformed with *MLL-AF9* AML were treated with 200 nM DPI, 20 mM oxamate, or their combination for 24 hours. Subsequently, 10^6 viable cells were transplanted into sublethally irradiated C57BL/6J mice, and Kaplan-Meier survival analyses were performed. All animal protocols were approved by Bioethics Committee of the University of Salamanca and by the Regional Government of Castile and León, and performed in accordance with institution guidelines.

Gene deletion

CYBB (NOX2 coding gene) deletion in THP-1 cells was achieved using CRISPR-Cas9 technology. The cell lines used in this study were previously characterized [2].

Statistical Analysis

Statistical significance (p-value <0.05) between groups was determined using Student's t-test and ANOVA for quantitative parametric variables, while the Mann–Whitney U and Kruskal–Wallis tests were employed for quantitative nonparametric variables. Overall survival (OS) curves were constructed using the Kaplan–Meier method, and the log-rank test was used to assess the statistical significance of differences between groups (p-value <0.05).

SUPPLEMENTARY FIGURES

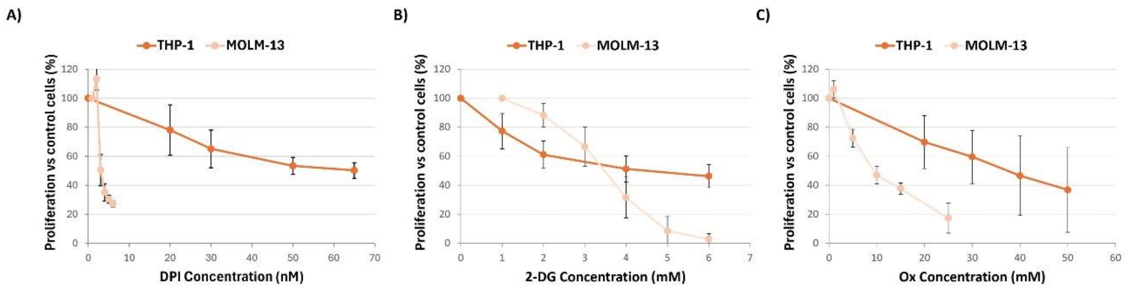

Figure S1. NADPH oxidase and glycolysis inhibitors reduce the proliferation of AML model cell lines. Curve-response growth of THP-1 and MOLM-13 cultured for 48 hours in the presence or absence of: A) diphenyleneiodonium (DPI), a common NADPH oxidase inhibitor; B) 2-Deoxy-D-Glucose (2-DG), a glucose analogue inhibiting hexokinase; C) oxamate, an inhibitor of lactate dehydrogenase (LDH).

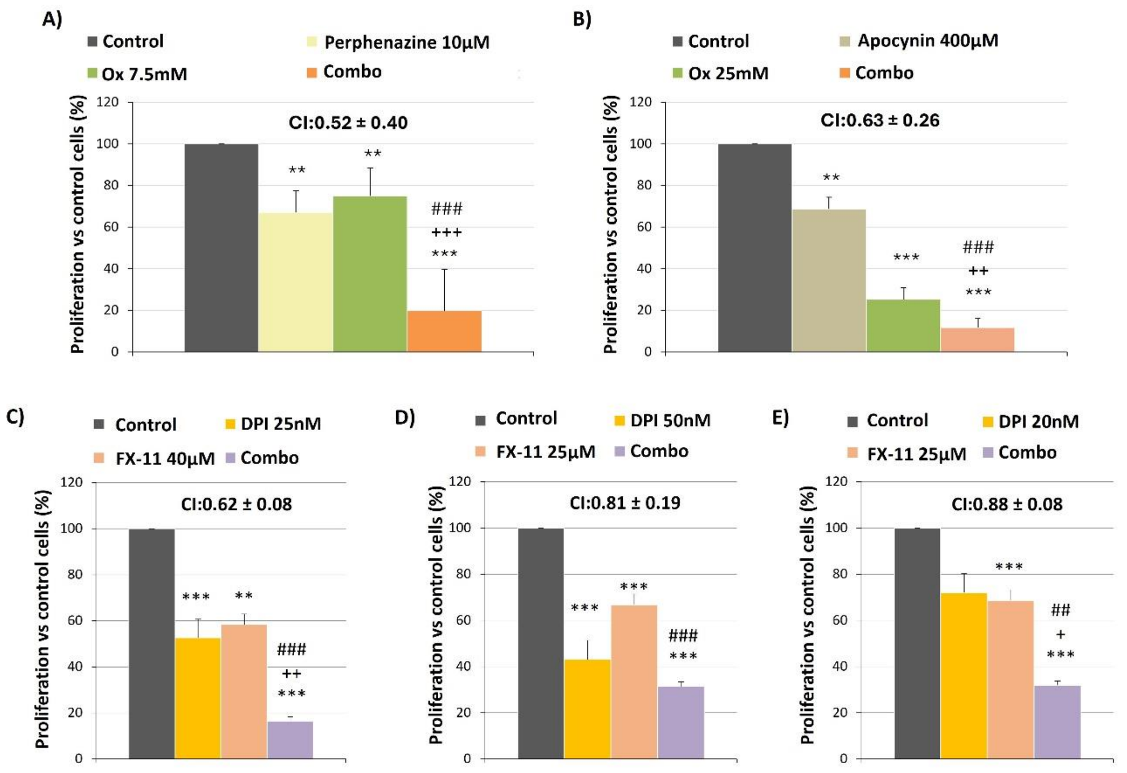

Figure S2. Alternative inhibitors of both, NADPH oxidases and LDH, also reduces the proliferation of AML model cell lines. AML cell lines were cultured for 48 hours in the presence or absence of different concentrations of LDH inhibitors (oxamate or FX-11), NOX inhibitors (DPI, perphenazine or apocynin) or their combination. A) Cell proliferation rate versus untreated control cells of the combination of Ox with perphenazine in HL60 cell line (n=5). B) Cell proliferation rate versus untreated control cells of the combination of Ox with apocynin in MOLM-13 cell line (n=5). (C-E) Cell proliferation rate versus untreated control cells of the combination of FX-11 with DPI in MOLM-13 (C), THP-1 (D) or NB-4 (E) cell lines (n=5). The combination index (CI) is indicated for each inhibitor combination. Results are shown in terms of the mean ± standard deviation. ***p<0.001, **p<0.01 and *p<0.05 reflect significant differences compared to untreated control cells. +++p<0.001, ++p<0.01 and +p<0.05 reflect significant differences compared to cells treated with NOX inhibitors, DPI, perphenazine or apocynin respectively. ###p<0.001, ##p<0.01 and #p<0.05 reflect significant differences compared to cells treated with metabolism inhibitor, Ox or FX-11 respectively.

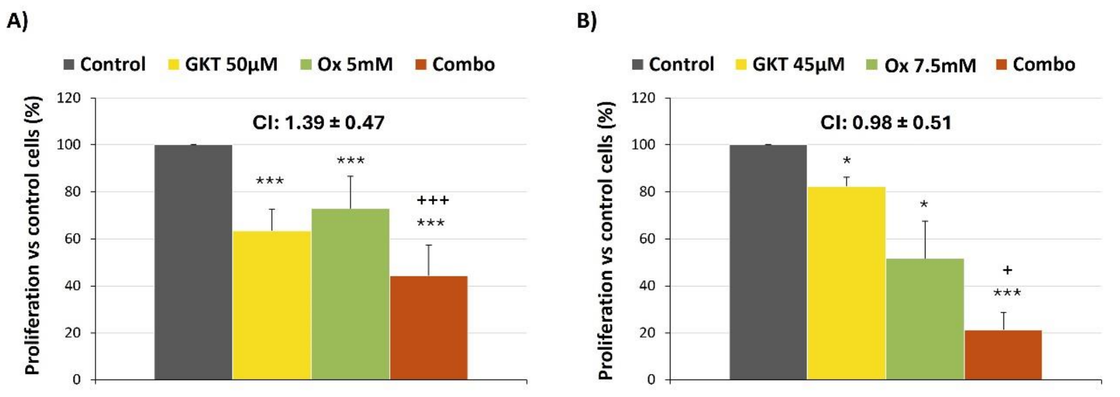

Figure S3. The combination of oxamate with a NOX1/NOX4-specific inhibitor does not exhibit synergy. AML cell lines were cultured for 48 hours in the presence or absence of different concentrations of LDH inhibitor oxamate, NOX isoform specific inhibitors GKT137831 (NOX1/NOX4 specific inhibitor) or their combination. (A-B) Cell proliferation rate versus untreated control cells of the combination of Ox with GKT137831 in HL60 (A) and MOLM-13 (B) cell lines. ***p<0.001, **p<0.01 and *p<0.05 reflect significant differences compared to untreated control cells. +++p<0.001, ++p<0.01 and +p<0.05 reflect significant differences compared to cells treated with GKT137831 alone. ###p<0.001, ##p<0.01 and #p<0.05 reflect significant differences compared to cells treated with Ox.

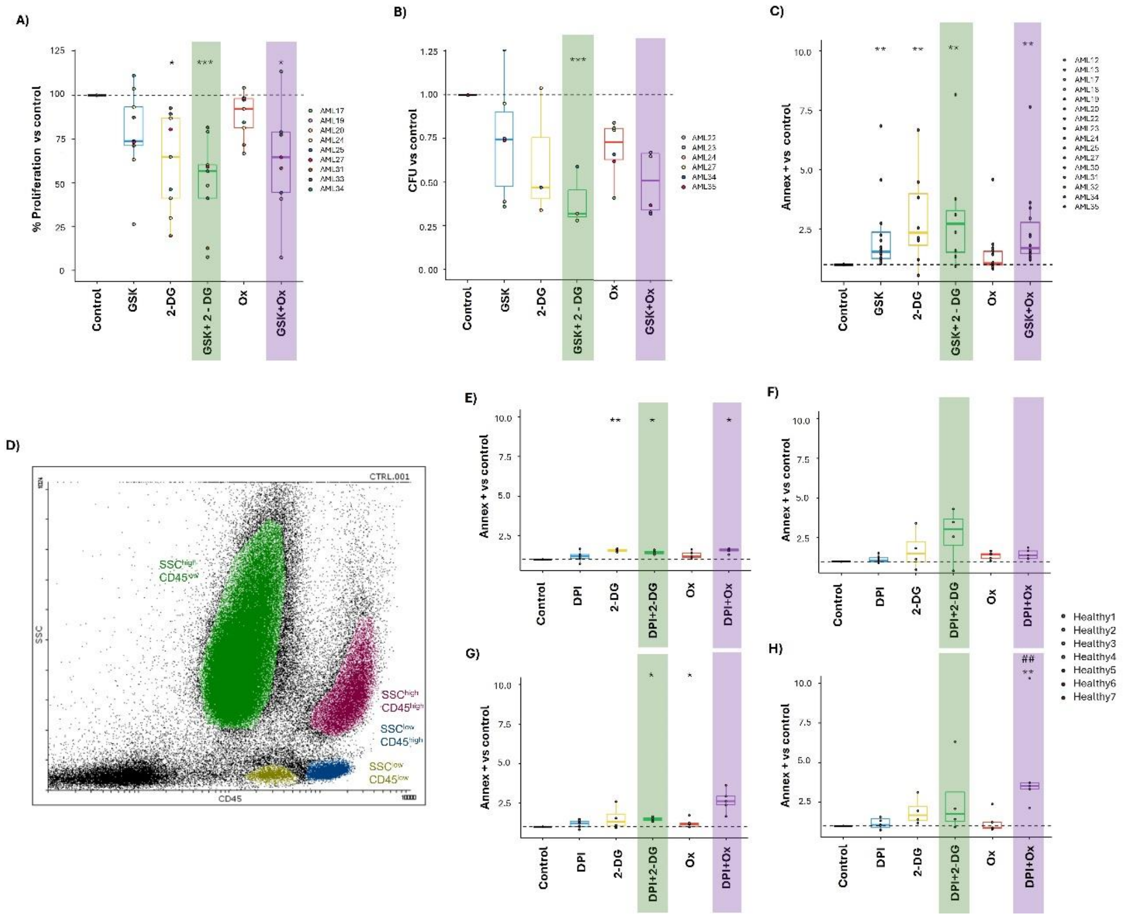

Figure S4. The combination of glycolysis inhibitors with GSK2795039, a NOX2-specific inhibitor, effectively impairs AML patient cells *in vitro* while demonstrating less harm to blood cell populations from healthy individuals. Mononuclear cells, extracted by ficoll gradient from bone marrow of different AML patients or healthy donors, were cultured at a density of 5x10^5 cells/ml for proliferation assays and 1x10^6 cells/ml for clonogenicity and cell viability assay for 48 hours in the presence of 2mM 2-DG, 10mM Ox and 100nM DPI, GSK2795039 100µM or their combination. A) Cell proliferation was analysed by MTT assay when 2-DG or Ox was combined with GSK2795039 in AML patients BM-MNC (bone marrow mononuclear cells). B) Normalization of the number of colony forming units (CFU) relative to control counted after culturing 10^4 mononuclear cells from different AML patients previously treated with 2-DG or Ox combined with GSK2795039 in 500 µl of semi-solid methylcellulose medium for two weeks was assessed. C) Cell death induction of

Annexin+ cells in the SSC^{low} CD45^{low} population was quantified by combining 2-DG or Ox with GSK2795039. D) Four populations can be distinguished in the dot plot derived from SSC-CD45 parameter discrimination: SSC^{low} CD45^{low} in gold, SSC^{high} CD45^{low} in green, SSC^{low} CD45^{high} in blue and SSC^{high} CD45^{high} in garnet. E-H) Cell death induction of Annexin+ cells in the SSC^{low} CD45^{low} (E), SSC^{high} CD45^{low} (F), SSC^{low} CD45^{high} (G) and SSC^{high} CD45^{high} (H) population was quantified when 2-DG or Ox was combined with DPI in healthy donor BM-MNC. All graphs are shown normalising the data to the control. **p<0.001, **p<0.01 and *p<0.05 reflect significant differences compared to untreated control cells. +++p<0.001, ++p<0.01 and +p<0.05 reflect significant differences compared to cells treated with NOX inhibitor, GSK2795039 or DPI, respectively. ###p<0.001, ##p<0.01 and #p<0.05 reflect significant differences compared to cells treated with metabolism inhibitor, 2-DG or Ox. 2-DG, 2-Deoxy-D-Glucose; Ox, oxamate.

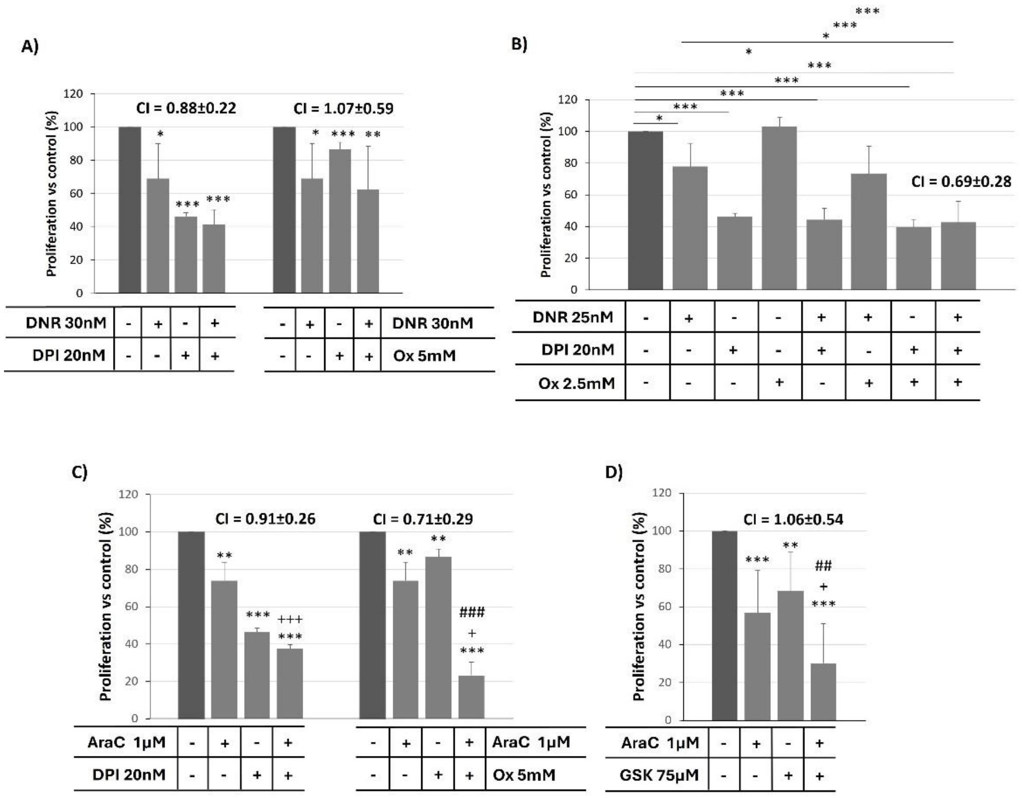

Figure S5. No interaction was observed with daunorubicin, but cytarabine showed positive interactions with DPI, GSK2795039, and oxamate. THP-1 cells were cultured to assess cell proliferation after 48 hours in the presence of daunorubicin (DNR) or cytarabine (AraC), Ox, DPI or GSK2795039, or their combination. A) Cell proliferation after treatment with the double combinations of DNR + DPI or DNR + Ox (n=5). ***p<0.001, **p<0.01 and *p<0.05 reflect significant differences compared to untreated control cells. B) Cell proliferation after treatment with the triple combination of DNR + DPI + Ox (n=7). ***p<0.001, **p<0.01 and *p<0.05 reflect significant differences. C-D) Cell proliferation after treatment with the double combinations of Ara-C + DPI and Ara-C + Ox (C) or Ara-C + GSK (D). ***p<0.001, **p<0.01 and *p<0.05 reflect significant differences compared to untreated control cells. +++p<0.001, ++p<0.01 and +p<0.05 reflect significant differences compared to Ara-C only treated cells. ###p<0.001, ##p<0.01 and #p<0.05 reflect significant differences compared to cells treated with DPI, Ox or GSK2795039 respectively.

REFERENCES

[1] Sardina JL, López-Ruano G, Sánchez-Abarca LI, et al. P22phox-dependent NADPH oxidase activity is required for megakaryocytic differentiation. Cell Death Differ 2010; 17: 1842–1854.

[2] Chou T-CC. Drug combination studies and their synergy quantification using the Chou-Talalay method. Cancer Res 2010; 70: 440–446.

[3] Parker JW. Flow cytometry in the diagnosis of lymphomas. Cytometry 1988; 9: 38–43.

[4] Eriksson M, Peña-Martínez P, Ramakrishnan R, et al. Agonistic targeting of TLR1/TLR2 induces p38 MAPK-dependent apoptosis and NFκB-dependent differentiation of AML cells. Blood Adv 2017; 1: 2046–2057.

[5] Miller PG, Al-Shahrour F, Hartwell KA, et al. InVivo RNAi Screening Identifies a Leukemia-Specific Dependence on Integrin Beta 3 Signaling. Cancer Cell 2013; 24: 45–58.

[6] Ijurko C, Romo-González M, García-Calvo C, et al. NOX2 control over energy metabolism plays a role in acute myeloid leukaemia prognosis and survival. Free Radic Biol Med 2023; 209: 18–28.
